# Assessment of Soil Sealing Management Responses, Strategies, and Targets Toward Ecologically Sustainable Urban Land Use Management

**DOI:** 10.1007/s13280-014-0511-1

**Published:** 2014-04-17

**Authors:** Martina Artmann

**Affiliations:** Department of Geography and Geology, Paris-Lodron-University Salzburg, Hellbrunnerstraße 34, 5020 Salzburg, Austria

**Keywords:** Soil sealing, Soil sealing management, Ecosystem services, Germany, Urban green, Urban planning

## Abstract

Soil sealing has negative impacts on ecosystem services since urban green and soil get lost. Although there is political commitment to stop further sealing, no reversal of this trend can be observed in Europe. This paper raises the questions (1) which strategies can be regarded as being efficient toward ecologically sustainable management of urban soil sealing and (2) who has competences and should take responsibility to steer soil sealing? The analyses are conducted in Germany. The assessment of strategies is carried out using indicators as part of a content analysis. Legal-planning, informal-planning, economic-fiscal, co-operative, and informational strategies are analyzed. Results show that there is a sufficient basis of strategies to secure urban ecosystem services by protecting urban green and reducing urban gray where microclimate regulation is a main target. However, soil sealing management lacks a spatial strategically overview as well as the consideration of services provided by fertile soils.

## Introduction

The ongoing urbanization is one of the main threats for sustaining ecosystems’ capability to supply ecosystem services to humans (MA 2005). Cities are characterized by a high degree of impervious surfaces and by continuous built-up areas (e.g., Turok and Mykhnenko 2007). Hence, urban growth promotes the increase in land take and soil sealing. Land take is understood as the conversion of open areas into built-up areas and can also include non-sealed areas such as gardens. Soil sealing is defined as the permanent covering of soil by completely or partly impermeable artificial material (Prokop et al. 2011). Sealing by urban gray infrastructure, which includes all forms of pavements and buildings (according to Breuste 2011), has especially negative impacts on the potential provision of ecosystem services. Soil sealing influences regulating services by increasing water surface runoff (Haase and Nuissl 2007) and microclimate regulation by increasing temperatures (Henry and Dicks 1987). It reduces provisioning services such as food production since fertile agricultural areas in particular get lost (Burghardt 2006). Furthermore, due to loss and fragmentation of habitats for flora and fauna, soil sealing has negative impacts on supporting services and is threatening urban biodiversity (Montanarella 2007). Moreover, the supply of cultural services is under pressure, since recreational areas within urban core districts are threatened by (re-)densifications (Niemelä et al. 2010).

Despite shrinking of the European population, a constant increase in impervious surfaces within the European Union can be observed (Prokop et al. 2011). The fact that there is a need to stop further soil sealing has already affected policies at the European and national level (EC 2012; EEA 2012). However, between 1990 and 2006 an increase of 8.8 % in artificial surfaces could be observed and, in 2006, 2.3 % of the European territory was sealed (Prokop et al. 2011). In Germany, a target was formulated which recommends the decrease of daily land take to 30 ha day^−1^ in 2020. This target seems difficult to reach since in 2010 still 77 ha day^−1^ were being taken for transport and settlement areas (Statistisches Bundesamt 2012). Neither a target on sealing reduction nor a standardized sealing monitoring exists in Germany. However, estimates show that between 46 and 50 % of transport and settlement areas are sealed (Breitenfeld 2009). Today, 5 % of the German territory is covered by impervious surfaces (Prokop et al. 2011).

Since cities in particular are characterized by a high degree of impervious surfaces, it is crucial to steer urban soil sealing in an ecologically sustainable way to secure urban ecosystems’ ability to sustain ecosystem services for their residents. Therefore, this paper investigates (1) which responses, strategies, and sub-targets can be regarded as being efficient toward ecologically sustainable management of urban soil sealing and (2) who has competences and should take responsibility to steer soil sealing? Germany was chosen as the study area as this is one of the most sealed countries within the EU (Prokop et al. 2011). Table 1 provides definitions of the main terms used and their relation to the research questions.

### Scales of Investigation and Study Area

The research integrates three scales, taking into account steering competences and addressees at the macro-, meso-, and microscale. At the macroscale regions (Region of Western Saxony/Region of Munich), federal states (Saxony/Bavaria) and the federal government (Germany) were considered. At the mesoscale, shrinking and growing cities with over 100 000 inhabitants in Germany were analyzed as it is assumed that the challenges cities face are especially complex due to the larger scale. Fourty-seven percent of European cities have a population of over 100 000 inhabitants (EC 2011). European cities are facing economic changes such as deindustrialization (Turok and Mykhnenko 2007), which offer cities the opportunity to re-use urban industrial wastelands to reduce further sealing. Moreover, European cities are confronted with social individualization, which leads to an increase in living space per capita. This hinders a reduction of land take (Haase et al. 2013) and decreases urban green areas (Kabisch and Haase 2013), which are essential for ecosystem service provision (Bolund and Hunhammar 1999). Growing and shrinking cities were differentiated because they face various challenges in urban management. Two case study cities were selected under specific selection criteria. Leipzig was selected between 1998 and 2008 as the highest increase in settlement and transport areas in Germany was recorded in the city despite the shrinkage processes. Munich was chosen due to a high increase in recreational areas between 1998 and 2008 and a high increase in population at the same time.

Leipzig is situated in Saxony, East Germany and has a population of 520 838 in 2012 (www.statistik.leipzig.de). Because of losing in economic importance in the 1960s, Leipzig experienced a high population migration. Despite processes of shrinkage, suburbanization, and urban sprawl could be observed, reaching their peak in the late 1990s (Haase and Nuissl 2010). Leipzig today is an example where both processes of shrinkage in the urban periphery and re-urbanization, especially in the urban core areas, can be found (Haase and Nuissl 2007). Previous studies on soil sealing development between 1997 and 2003 showed that sealing efficiency decreased during sealing at the urban fringes by commercial and industrial sites and low density residential areas. In total, an increase in sealed surfaces of 2.84 % could be observed and in 2003, 27 % of the area was sealed (Artmann 2013a).

With a population of 1.4 million (2011), Munich is the third largest city in Germany. Munich is characterized by a high immigration pressure as the population increased by over 200 000 residents between 1990 and 2010. Further population growth of 100 000 residents is projected by 2020. Moreover, Munich can be characterized by an urban re-organization due to the privatization of the German Railway System and the closing of barracks that were used for new residential and recreational areas. Compared to Leipzig, this supported a low increase of sealing, 0.4 % between 1998 and 2011 and in 2011, 36 % of the area was sealed. However, no further wastelands are available now and new residential areas should be built by further densification which threatens the loss of ecosystem services, especially in the urban core areas where green areas are already under pressure due to their small sizes and low per capita supply (Artmann 2013a). At the microscale, the civic society (NGOs and residents) and practitioners of relevance for soil sealing management (investors and (landscape) architects) were considered.

## Materials and Methods

### Definition of Set of Instruments and Its Steering Competences and Addressees

**Fig. 1:**
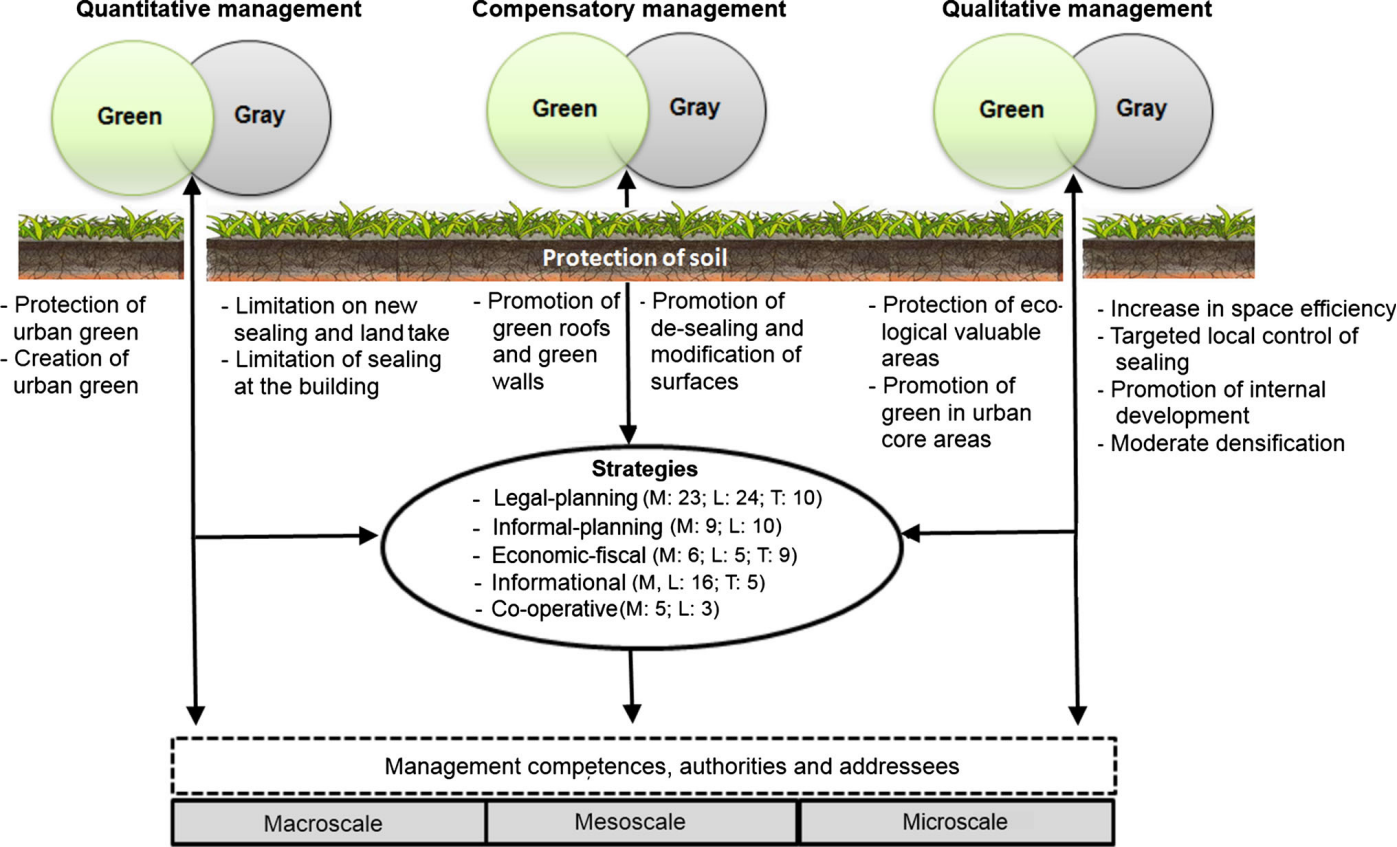
Framework for multi-scale analyses of soil sealing management instruments (*M* Munich, *L* Leipzig, *T* in theory discussed responses) (icon for soil by Osada 2011)

To analyze the efficiency of soil sealing management strategies and sub-targets toward an ecological urban sustainable development (research question 1), sets of instruments considering a holistic soil sealing management approach were first defined. Sets of instruments were defined since German policy assumes that the 30-ha target can only be achieved by a mix of instruments (Deutscher Bundestag 2004). A holistic soil sealing management approach includes quantitative, qualitative, and compensatory management of urban gray and urban green as well as the protection of soils as the basis of urban gray and green. These steering dimensions are defined as sub-targets in this paper (Table 1) and were derived from a spatial analysis of soil sealing development (Artmann 2013a). For assessing how these sub-targets can be achieved, strategies were identified by reviewing planning documents and literature. For soil sealing, relevant strategies include legal-planning (including laws and informal planning), economic-fiscal (e.g., subsidies, taxes), co-operative (e.g., regional or sectoral co-operations), and informational strategies (e.g., spatial monitoring, awareness raising, improving know-how) (Artmann 2013a). Specific instruments (named as responses) of each strategy were selected and assigned to the sub-targets via criteria (see Fig. 1).

**Table 1:** Glossary of main terms and their relation to the research questions

Term	Definition
Efficiency	Criterion of assessment which describes to which degree a response is suitable to achieve an objective in a certain way. The definition of an ecologically efficient soil sealing management approach is provided in Table 2
Response	Specific instrument which aims to steer soil sealing (e.g., a specific law such as the building code). This paper assesses the efficiency of ecological sustainable responses
Strategy	Strategy is understood as the sum of responses addressing the same types of steering. Within this study legal-planning, informal-planning, economic-fiscal, co-operative and informational strategies are investigated. The efficiency assessment of strategies is based on the assessment of responses which are assigned to strategies (see Fig. 1)
Sub-targets	Sub-targets define what has to be steered spatially in the course of a holistic soil sealing management approach. These targets relate to steering urban green (open land such as forests and agricultural land, recreational areas), gray (built-up areas and artificial material) and soil (land and substrate). Urban green and gray can be steered quantitatively (reduction of new sealing and land take, protection of green areas), qualitatively (promotion of internal development and space efficient building forms, protection of green areas with high ecological performance). Moreover, existing sealed areas can be compensated by de-sealing or greening roofs (see also Fig. 1). The efficiency assessment of sub-targets is based on the assessment of responses which are assigned to the sub-targets
Actors	Actors of soil sealing management refer to administrative units and communities responsible for developing and implementing strategies in the course of a holistic soil sealing management. The responses selected are assigned to groups of actors of different management scales to prove who is responsible for soil sealing management and to which degree (see Fig. 1)

The selection of responses was done by reviewing laws as well as local-planning documents (zoning, landscape, sectoral, and informal plans), scientific literature and projects (such as REFINA, Research for the Reduction of Land Consumption and Sustainable Land Management), local initiatives and by conducting expert interviews including experts from the departments of planning, environmental reporting, environmental protection, urban redevelopment, and construction as well as NGOs, real estate agents and research. The responses selected should have relevance for steering soil sealing, land take and land use, urban green areas, and soil as part of a holistic soil sealing management. The focus of this paper is on responses in use. However, in further studies, theoretically discussed responses will be included. In total, 93 responses in practice and 24 theoretically discussed responses were identified and assigned to the sub-targets, whereas a response can be assigned to more sub-targets but only to one strategy. The number of responses selected per strategy is shown in Fig. 1. To analyze the main management authorities and addressees (research question 2), the selected responses were assigned to the macro- (state government, federal states, region), meso- (city level), and microscale (civic society, practitioners) by identifying who has the power to develop a response (authority) and who is responsible for implementing it (addressees) (see Fig. 1) (Artmann 2013b).

### Indicators to Assess Strategies Toward an Ecologically Sustainable Soil Sealing Management

The efficiency assessment of strategies and spatial sub-targets toward ecologically sustainable management was based on indicators. Indicators are useful as they support policy and decision makers by providing comprehensible and quick information on consequences of steering actions on the environment (Pulles and van Harmelen 2004). The indicators were derived by developing hypotheses of an ecologically sustainable development based on structured expert interviews in Leipzig and Munich, literature review and analyses of impacts by soil sealing on ecosystem services provisioning (Artmann 2013c). The indicators should reflect impacts of sealing on the urban ecosystem and ecosystem service provisioning as well as framework conditions for ecologically sustainable management.

#### Indicators on Impacts by Soil Sealing on Ecosystem Service Supply

Ecosystem service supply strongly depends on land use. Therefore, indicators that assess the supply of ecosystem services should be sensitive to land use change (Larondelle and Haase 2013). Following this, land use policy steering urban soil sealing in an ecological sustainable manner should be aware of impacts on ecosystem service provision by soil sealing. This target becomes even more crucial as cities face global climate hazards (Bulkeley 2013) which are intensified by soil sealing. Moreover, according to experts in Leipzig and Munich, the increasing importance of “soft” location factors, including sufficient supply of recreational areas, improve the consciousness of impacts by sealing (Artmann 2013c). Recreational areas should offer characteristics such as “wilderness” or a “rich variety of species” (Herzele and Wiedeman 2003) and can thus be managed like urban forests. In contrast, urban parks are more managed (Bolund and Hunhammar 1999) but also provide physical and psychological well-being for urban dwellers (Chiesura 2004). Besides public green spaces, private green areas such as gardens and allotments are crucial for supporting urban biodiversity and for experiencing urban wildlife (Goddard et al. 2010). Spatial analyses of impacts on soil sealing in Leipzig between 1997 and 2003 showed that, in particular, soils of high quality were used for transport and settlement areas as part of the suburbanization processes (Artmann 2013a). The loss of valuable soils by sealing is crucial as fertile soils affect vital processes and functions such as nutrient cycling processes, seed dispersal, or pollination, which yield ecosystem services (Boyd and Banzhaf 2007). According to an expert of the Saxon State Office for the Environment, Agriculture, and Geology and a scientific expert, improved protection of agricultural areas could be promoted by stressing the importance of agricultural land for nutrition. To secure ecosystem services, the obligatory integration of ecological aspects and reduction of further sealing into decision making is crucial (Artmann 2013c).

#### Indicators on Framework Conditions for a Sustainable Ecological Development

Spatial analyses in Munich on drivers of urban soil sealing between 1998 and 2011 showed that the main drivers of sealing were transport areas, which increased especially at the urban fringes (Artmann 2013a). In general, urban sprawl increases the distances between working and living and therefore the need for roads, which leads to an increase in the use of cars, energy consumption, and traffic emissions (de Ridder et al. 2008). Therefore, reducing private motorized traffic can support a reduction in sealing and at a larger scale also in energy consumption and air pollution (Artmann 2013a). Soil sealing management should therefore also include a spatial strategic overview and consider impacts by urban land use changes on distant rural places, also termed urban land teleconnections (Seto et al. 2012). Besides the spatial scale, a temporal hypermetropia is vital as the definition of sustainability in the Brundtland Report emphasizes achieving present development in a way which ensures that future generations can also meet their own needs.

### The Assessment Process

The assessment of soil sealing management responses, strategies, and spatial targets toward an ecologically sustainable urban sealing management approach was based on a multi-attribute decision method (MADM) using an analytical hierarchy process (AHP). The MADM allows a comparison between several alternatives by using a set of indicators and therefore supports decision making (Zanakis et al. 1998). Within an AHP, one form of MADM, alternatives are compared in pairs including decision makers’ preferences (Saaty and Vargas 2012). The assessment process included three steps: (1) assessment of importance of indicators, (2) content analysis of responses, and (3) evaluation of analyses results. More information on the method developed for Response-Efficiency-Assessment (REA) can be found in Artmann (2013b).

The assessment of the importance of indicators (step 1) was done by involving decision makers of the mesoscale responsible for urban development and planning, brownfield management, urban green management and nature conservation, soil sealing monitoring, urban renewal, and urban policy. In an online survey, the decision makers were asked to evaluate the importance of the indicators on a Likert Scale between 1 and 9, where 1 stood for not important and 9 for very important (see e.g., Mendoza and Prabhu 2000). The weighting factor *W*_I_ represents the mean value of the assessment (Table 2). The evaluation of the responses was carried out via a deductive content analysis (step 2), whereas the indicators served as a categorization matrix and were use to prove hypotheses developed before the analysis (Elo and Kyngäs 2008). Laws, planning documents, statements of initiatives, and co-operations were read carefully and data corresponding to the indicators excerpted. The excerpted passages were coded according to the indicators’ assessment score (IS) for each sub-target (Table 2).

**Table 2:** Indicators, assessment scores, and importance of indicators for assessing the efficiency of strategies toward ecologically sustainable soil sealing management (ES, Ecosystem service)

Indicator	Indicator assessment score IS (between 1 and 9)	Weighting factor *W*_I_ Munich (*N* = 13)	Weighting factor *W*_I_ Leipzig (*N* = 13)
Securing, improvement and development of habitats for flora and fauna	9: Protection of ES by securing green areas or soils/by reducing sealing is clearly stated as target interlinked with benefits derived by protection/reduction (e.g., reducing further sealing to protect habitats for flora and fauna and to improve contact to nature for residents) 7: Importance of ES/function is mentioned but not directly linked to targets such as reduction of further sealing/protection of green/soils (e.g., green areas are important for flora and fauna; sealed surface increase urban heating) 4: Protection/importance of aspects related to ES/functions are mentioned but they are not directly linked to benefits/harm by green areas/soils or sealing (e.g., measures for climate adaptation have to implemented, such measures could also integrate technical solutions) 1: ES not mentioned	6.85	7.00
Improving surface water run-off		7.08	8.13
Improving climate adaptation (decrease heat emission, increase carbon binding)		7.23	7.75
Improving private recreational areas (gardens, courtyards)		6.69	7.19
Improving public green areas (more managed areas such as parks)		6.69	6.44
Improving recreational areas (less managed, e.g., forests, landscape parks)		6.62	6.44
Protection of agricultural areas for food production		5.54	7.00
Protection of ecologically valuable fertile soils and their functions		7.00	7.44
Reducing motorized private transport	9: Demand for reduction of private motorized transport/development of public transport is mentioned related to the reduction of sealing/protection of green/soils (e.g., the development of public transport is crucial to promote urban internal development) 7: Demand for reduction of motorized private transport/development of public transport is mentioned but not interlinked to targets for reducing sealing/protection of green/soils (e.g., further transport areas increase sealing) 4: Demand for reduction of impacts by motorized private transport/development of public transport are mentioned but not linked to reducing sealing/protecting green areas (e.g., a decrease in motorized traffic reduces the air and noise pollution) 1: Demand for reduction of motorized private transport/development of public transport is not mentioned	6.54	7.50
Spatial strategic overview	9: supra-regional view; 7: regional view; 4: city view; 1: less than city view/no spatial view	6.85	6.19
Temporal hypermetropia	9: >20 years; 7: 20–11 years; 5: 10–6 years; 3: 5–1 year; 1: no temporal course mentioned; 9: Integration ecological aspects before project implementation; 5: Integration ecological aspects during project implementation; 1: Integration ecological aspects after project implementation	6.46	7.31
Priority setting: Obligation for considering ecological aspects/reducing sealing or possibility of consideration	9: Reduction of further sealing/integration of ecological aspects is obligatory; 5: Reduction of further sealing/integration of ecological aspects is demanded but not binding as part of a weighing-up process with other aspects; 1: Ecological aspects are not mentioned at all	6.62	7.50

Afterward, the response efficiency (RE) was calculated for each response R of a strategy S separately for each sub-target ST (step 3): all indicator scores IS derived by the responses R within the spatial sub-targets ST (0)–(VI) were summed up and divided by the number *N* of responses R per strategies S reviewed. The sub-targets stand for (see also Fig. 1) (0) protecting soil; (I) quantitative steering urban gray; (II) quantitative steering urban green; (III) qualitative steering urban gray; (IV) qualitative steering urban green; (V) compensation measures for urban gray; and (VI) compensation measures for urban green. The quotient was multiplied by the weighting factor *W*_I_ (see Table 2):

$$ {\text{RE}}_{{{\text{ST}} - {\text{S}}}} = \left( {\frac{{\sum_{I = 1}^{12}   {\text{IS}}_{{{\text{R}} - {\text{ST}}}}  }}{{N_{{{\text{R}} - {\text{S}}}} }} \times W_{\text{I}} } \right) $$

The results are provided in % of the maximal reachable weighted score *W*_I_ per strategy S. For analyzing the most efficient strategy and spatial sub-targets toward an ecological sustainable soil sealing management, the percentage scores reached per strategy and sub-target were summed up, and the mean value for the strategies (5 strategies) and spatial targets (7 targets) was calculated.

## Results

**Fig. 2:**
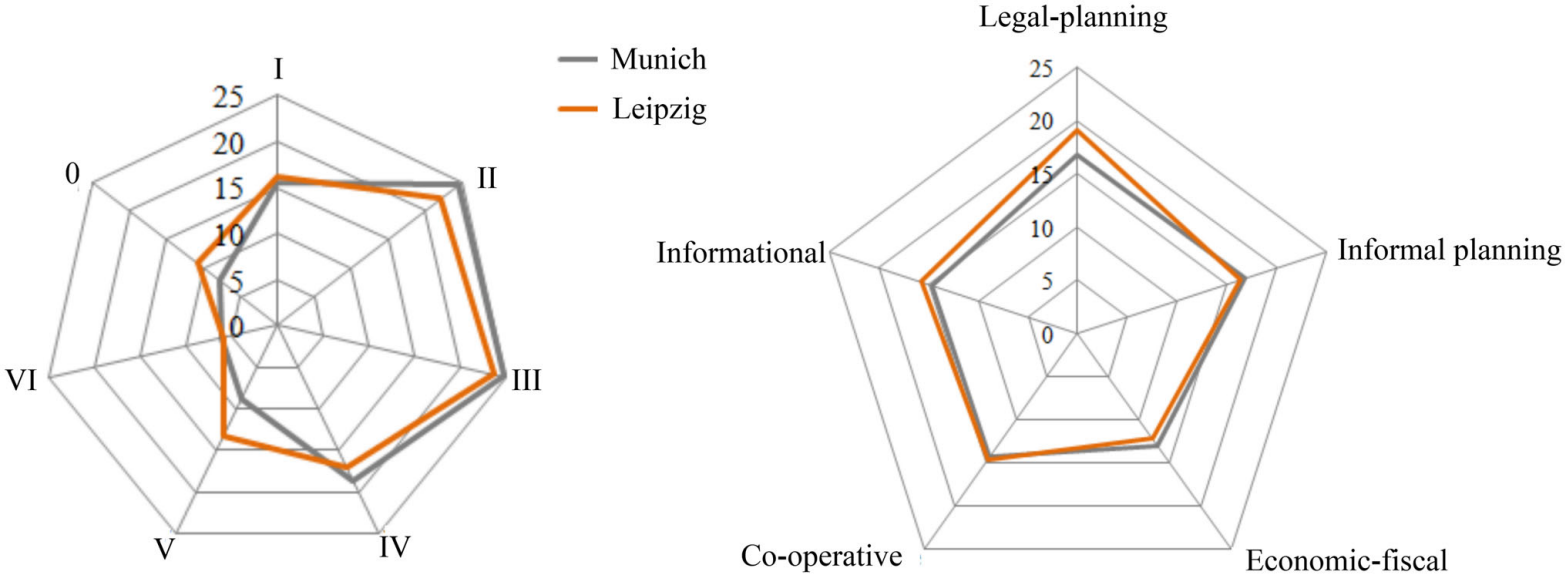
Spidergrams comparing the efficiency of spatial targets (*left*) and strategies (*right*) for ecologically sustainable soil sealing management (in %) in Leipzig and Munich. Sub-targets for steering soil sealing: *0* protecting soil; *I* quantitative steering urban gray; *II* quantitative steering urban green; *III* qualitative steering urban gray; *IV* qualitative steering urban green; *V* compensation measures for urban gray; *VI* compensation measures for urban green

Figure 2 summarizes the average efficiency of strategies and spatial sub-targets toward an ecologically sustainable management as part of a holistic soil sealing management approach. In Munich and Leipzig, most of the responses analyzed focus on quantitative protection of urban green and qualitative steering of urban gray promoting infill development. Ecological arguments for infill development are a reduction of fragmentation of habitats, reduction of traffic, and protection of agricultural areas at the urban fringes. The protection of soil and the creation of green roofs are less often included in the reviewed responses. In Leipzig, de-sealing and dismantling measures are mentioned more often than in Munich especially in legal-planning and informal-planning documents to reduce transport areas, to adapt to climate change, and to improve recreational areas by demolishing buildings in highly densely built-up areas.

**Fig. 3:**
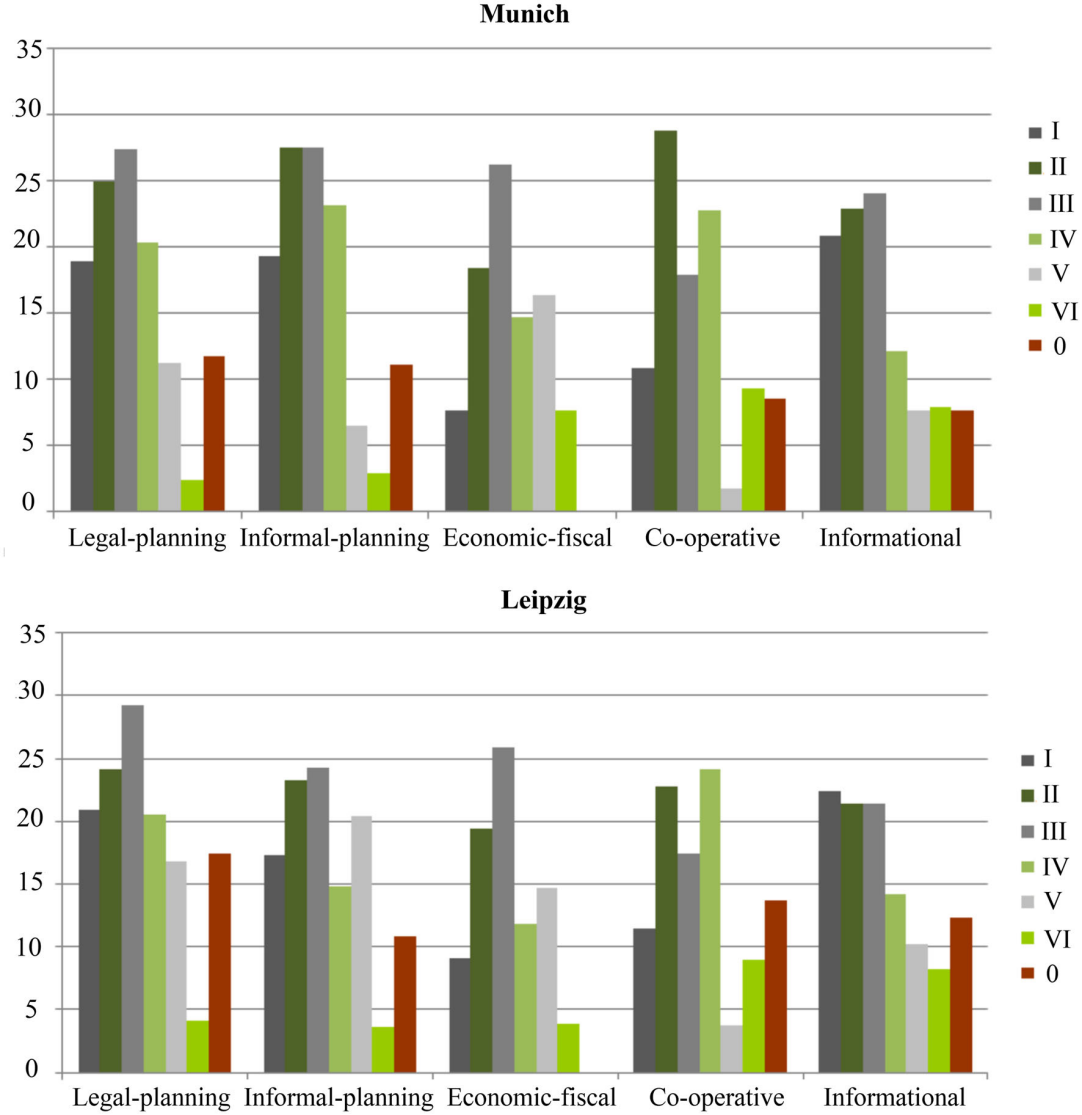
Efficiency of strategies to steer sub-targets of soil sealing management in Munich and Leipzig (in %). Sub-targets for steering soil sealing: *0* steering soil; *I* quantitative steering urban gray; *II* quantitative steering urban green; *III* qualitative steering urban gray; *IV* qualitative steering urban green; *V* compensation measures for urban gray; *VI* compensation measures for urban green

In Leipzig, as well as in Munich, steering by legal and informal planning seems to be the most efficient strategy followed by co-operative and informational strategies. The focus of legal and informal planning is especially on the promotion of urban infill development in course of a qualitative management of urban gray (Fig. 3). This sub-target is supported by the German Building Code (Baugesetzbuch) that allows a faster and more flexible realization of infill development waiving an environmental impact assessment. The aim is to protect natural areas and their fragmentation at the urban fringes. The quantitative reduction of further sealing is also demanded by the soil protection clause (Bodenschutzklausel), integrated in the German Building Code (Baugesetzbuch). Moreover, the open space plan of the City of Munich, the landscape plan of the City of Leipzig or the Regional Development plan of Bavaria and Saxony demand the reduction of further sealing, especially to improve microclimate regulation.

Economic-fiscal steering seems to be less efficient. This becomes especially obvious when looking at the economic-fiscal steering of soils for which no response could be identified (see Fig. 3). However, fiscal steering especially supports the promotion of inner development and the re-use of brownfields with the support of subsidies as well as the supply and quality of recreational areas. On the mesoscale, the city of Munich provides financial support to practitioners and residents for de-sealing and greening roofs to improve the infiltration of surface water runoff and microclimate regulation as well as to improve living quality in the highly sealed city of Munich. Within a co-operative sealing management strategy, the focus in Leipzig is on the quantitative and qualitative steering of urban green which is especially supported by the regional co-operation Green Ring Leipzig (Grüner Ring Leipzig) where green areas are to be protected, established, and interlinked to protect agricultural areas and their fertile soils for food production, the development of parks for recreation or forests for improvement of biodiversity. Soft strategies such as co-operative and informational responses especially support greening roofs to protect ecosystem services. This results especially from the transfer of know-how about ecologic advantages of green roofs through brochures. As part of a participatory survey on the living quality in Munich residents demand a reduction of traffic, also in connection with the de-sealing of streets and the creation of green areas for recreation. The reduction of motorized traffic is also one sustainability target in Leipzig but focuses more on consequences by traffic on air pollution rather than on the space taken by cars. Moreover, within the sustainability targets of Leipzig, a reduction of further sealing is demanded. However, in Leipzig, no sealing monitoring exists as an informational strategy which could prove the target achievement. In Munich, monitoring of sealing exists but no quantitative targets exist corresponding to the monitoring.

**Fig. 4:**
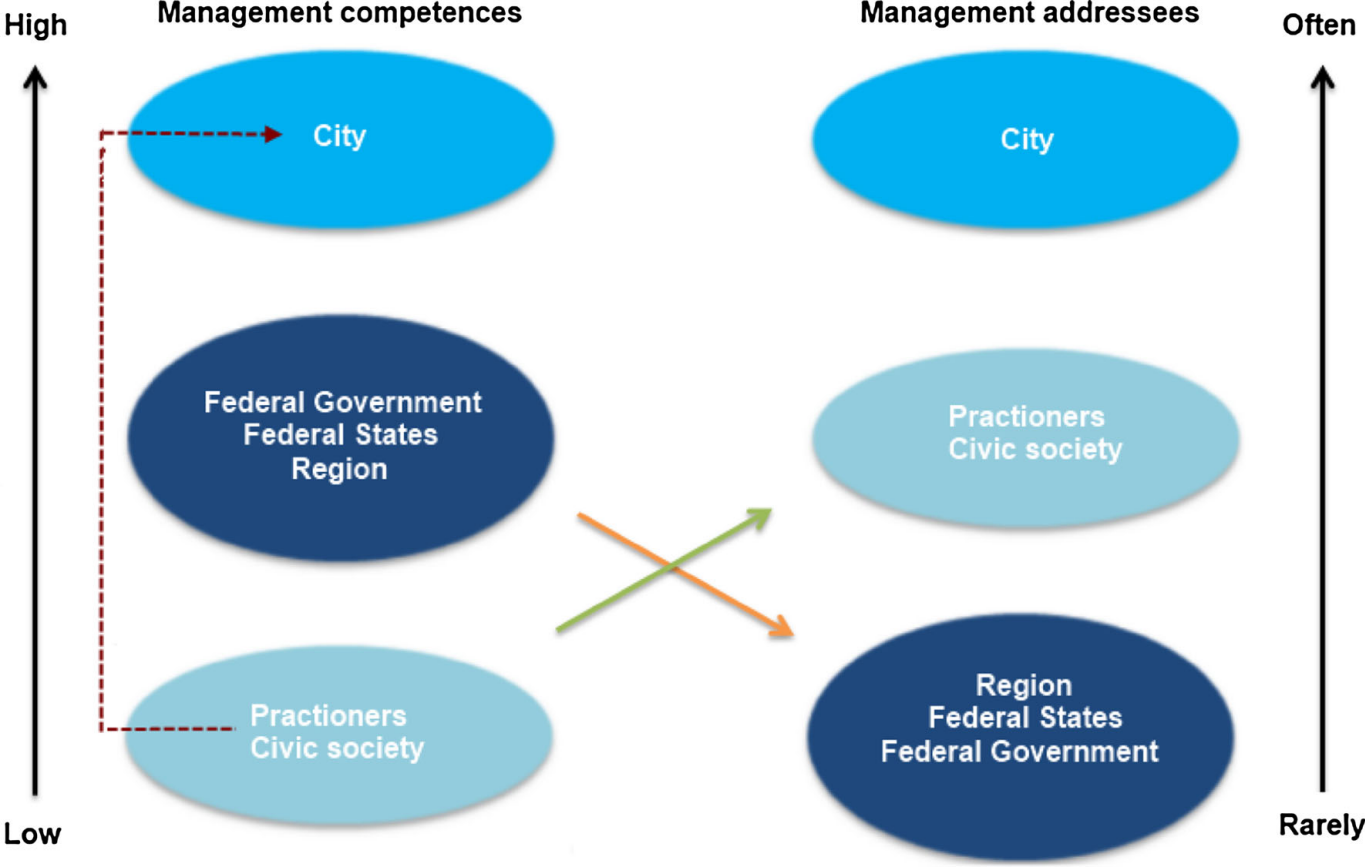
Management competences and addressees at the macro-, meso- and mircoscale based on the response analyses. The *green arrow* shows that actors at the microscale have less competence and are more often addressed for reducing soil sealing but influence the mesoscale (see *red arrow*). The *orange arrow* indicates that the macroscale has more competence but is less often addressed to implement any soil sealing measures

The analyses of management competences and addressees showed that most of the reviewed responses are developed at the mesoscale, and therefore the city level has the highest competence to take appropriate steps to manage urban soil sealing (Fig. 4). The competence especially includes the quantitative (31 responses) and qualitative steering (30 responses) of urban gray. The qualitative steering of urban gray is also the most often addressed steering target by the state government (16 responses) and federal states (20 responses). However, compensation strategies, for instance, for urban green (state government: 5 responses, federal states: 4 responses) are more rarely developed at the macroscale but more often at the city level (19 responses). At the same time, the cities′ policy and planning departments have to take responsibility to set the sub-targets through informal plans, monitoring systems, goals, co-operations between sectors as well as by putting laws stated by the state government into practice and acting as a role model to stop further loss of ecosystem services. Cities are especially addressed with respect to the implementation of strategies for qualitative steering of urban gray (28 responses) and quantitative steering of urban green (21 responses).

According to the review of responses, the microscale has developed responses less often as part of sealing management but is more important to put responses into practice. Therefore, the success of strategies and responses set by the city also depends on their steering potential on the microscale. For instance, subsidies for greening walls or taxes for waste water removal have to be high enough that residents implement such compensation measures. Informational strategies like brochures or consulting addressing the microscale could help to show up ecological and financial advantages of such actions.

## Discussion

### Efficiency of Soil Sealing Management to Secure Ecosystem Services

The evaluation of the responses showed that almost all strategies integrate ecological aspects as part of sealing management, which is also crucial in the course of global environmental change (Grimm et al. 2008). The importance of climate change within sealing management is also shown by the experts’ highly ranked importance of indicators on improving surface water run-off and climate adaptation (Table 2) as well as the ranking of the most important indicators of the content analysis (Table 3). The adaptation to climate change could, according to the reviewed responses, especially be achieved by a quantitative steering of urban green. The positive effect of urban green on the microclimate has been well investigated in a range of studies (e.g., Gill et al. 2007; Jo and McPherson 2001). In contrast, the study showed that ecosystem services provided by fertile soils are less integrated in the responses reviewed. This might also occur due to the lack of scientific studies on soil and its provision of ecosystem services (see review within this special issue, Haase et al. 2014).

**Table 3:** Ranking of the three most important indicators per strategy for protecting ecosystem services in course of soil sealing management

Rank	Legal-planning		Informal-planning		Economic-fiscal		Informational		Co-operative	
	M	L	M	L	M	L	M	L	M	L
(1)	Climate	Climate	Climate	Climate	Water	Water	Climate	Climate	Climate	Less managed
(2)	Water	Water	Water	Less managed	Climate	Habitat	More managed	Water	Habitat	More managed
(3)	Habitat	Habitat	Less managed	More managed	More managed	Climate	Less managed	Less managed	Less managed	Habitat

*M* Munich, *L* Leipzig, *climate* improving climate adaptation, *water* improving surface water run-off, *habitat* securing of habitats for flora and fauna, *more managed* improving public green areas (more managed), *less managed* improving recreational areas (less managed)

Moreover, the results demonstrated that the protection of soil by economic-fiscal strategies is missing in the case study cities in Germany. In other European countries, for instance, in Bulgaria or Poland, sealing of agricultural land is linked to a fee, the size of which depends on the quality of soil converted (EC 2012). However, although legal- and informal-planning strategies seem to be the most efficient, it cannot be confirmed within this study that a mix of economic-fiscal and land use planning instruments seems to be effective in reducing land consumption (Nuissl and Schroeter-Schlaack 2009), at least for steering soil sealing in an ecological manner.

The difference between the case study cities regarding the efficiency of steering was carried out by comparing the efficiency of spatial targets between Leipzig and Munich. The evaluation indicates the higher importance of de-sealing measures in Leipzig than in Munich due to high degrees of vacancy and brownfields that have arisen in the periods of shrinkage. In general, de-sealing is a chance to develop urban green areas, especially for Eastern European cities (Kabisch and Haase 2013). By investigating the development of urban green in 202 European cities, Kabisch and Haase (2013) further showed that an increase in living space per capita and in the number of smaller households hampers the reduction of further land take. Therefore, also practitioners and civic society need to be efficiently addressed to steer urban soil sealing; this also has been proven in this paper.

### Framework Conditions of Ecologically Sustainable Soil Sealing Management

Indicators reflecting framework conditions for an ecologically sustainable soil sealing management approach showed that a temporal hypermetropia mainly achieves high scores for the indicators. Moreover, the majority of the responses reviewed considered at least ecological aspects but mostly only within a city view (Table 4).

**Table 4:** Ranking of indicators per strategy regarding framework conditions for ecologically sustainable steering of soil sealing

Rank	Legal-planning		Informal planning		Economic-fiscal		Informational		Co-operative	
	M	L	M	L	M	L	M	L	M	L
(1)	Temp.	Temp.	Temp.	Temp.	Temp.	Temp.	Spat.	Spat.	Oblig.	Oblig.
(2)	Oblig.	Oblig.	Spat.	Oblig.	Oblig.	Spat.	Temp.	Temp.	Temp.	Spat.
(3)	Spat.	Spat.	Oblig.	Spat.	Spat.	Red.	Oblig.	Oblig.	Spat.	Red.

*M* Munich, *L* Leipzig, *temp.* temporal hypermetropia, *oblig.* obligation for considering ecological aspects/reduction of sealing, *spat.* spatial strategic overview, *red.* reducing motorized private transport

Although there is a call that urban sustainability needs to consider planetary stewardship (Seitzinger et al. 2012), the review of the responses confirmed that urban policies neglect that urban development depends on the hinterland and its ecological and economical services (Rees 1992). For instance, experts in the boomtown of Munich evaluated that the protection of agricultural land for food production is less important than supporting regulating and cultural ecosystem services (Table 2). Also the review of strategies in both case study cities showed that the protection of this service is rarely implemented. However, the loss of agricultural land by land consumption means that food has to be transported into the cities, which might promote sealing for roads and increase air pollution by traffic, which then has global impacts. Moreover, the high space demand for motorized traffic is neglected by the responses reviewed. This shows the need to improve the know-how by scientists and decision makers about complex impacts caused by urban soil sealing and loss of open areas through urban land teleconnections (Seto et al. 2012).

### Limits and Strengths of the Study

The evaluation of soil sealing management strategies and its efficiency toward an ecologically sustainable management approach was carried out by coding the results of the qualitative content analysis through numeric classes. This meant that weaknesses of a quantitative analysis, such as not seeing behind the scene of words and their meanings in a greater context (Selltiz et al. 1959), could be reduced. As the coding of the results is very context-sensitive, the process requires careful reading of the materials. Therefore, the content analysis was repeated twice. By using indicators and coding, the results to compare strategies and spatial sub-targets with each other, the approach presented in this paper complements the qualitative and less systematic analysis of best practice examples for soil sealing management by the European Commission (EC 2012). However, a pairwise ranking of each response by experts, like in a traditional AHP, was not done due to the high amount of responses studied; but at least experts were involved in developing the indicators and assessing their importance.

Limits in this study occurred through the selection of responses as it cannot be guaranteed that all responses handling soil sealing in the case study cities have been included. However, as experts were consulted in the identification of important responses and several studies on urban soil sealing and land take were revised, it can be assumed that especially legal-planning, informal-planning, and economic-fiscal strategies are complete as these provide the basis for urban development. However, informational and co-operative strategies might be incomplete as a range of small local initiatives could exist, such as civic greening communities (Bendt et al. 2013), which were not integrated separately into the study. However, the most important co-operations like the green ring Leipzig were elaborated. Further research is necessary to investigate to which degree such local greening initiatives support, for instance, the protection of urban green areas and the stewardship of ecosystem services, as undertaken by Bendt et al. (2013).

### Importance of Findings for Research on Ecosystem Services

Although responses for reducing sealing and protecting green for the provisioning of ecosystem service were identified, none of these mentioned the term “ecosystem services.” This has been shown in a study in Finland where most of urban land use planning actors were not familiar with the concept of ecosystem services although aspects of it were included in land use planning (Niemelä et al. 2010). Therefore, a closer co-operation between science and practice seems crucial to promote the concept of ecosystem services. This might improve a comprehensive understanding of municipalities and their inhabitants regarding the ecosystem and the benefits they derive from it for their well-being (Daily et al. 2009; Niemelä et al. 2010).

Findings of the study also showed that the qualitative steering of soil sealing is primarily understood as the promotion of infill development and densification neglecting that a sufficient supply of ecosystem services is also crucial for living quality in urban core areas as urban ecosystem services should be provided where they are consumed (Bolund and Hunhammar 1999). In this regard, the ecosystem service approach can provide decision support for policies to identify which green areas should be protected from further sealing and where sealing would be acceptable by assessing the supply and demand of ecosystem services. However, further research is necessary to provide standardized methods and indicators for planning and policy assessment, which are practical, applicable, comprehensive, credible, sensitive to changes in land management as well as temporarily and spatially explicit (van Oudenhoven et al. 2012).

## Conclusion

The paper introduced a new analytical approach to assess and compare strategies and spatial sub-targets to secure ecosystem services using the example of soil sealing management. It contributes to a clearer understanding about which ecosystem services are considered by planning and policy to be threatened through soil sealing and land consumption and which have to be secured by protecting urban green and soils. It could be shown that challenges as a result of climate change such as improvement of microclimate regulation and reduction of floods are the most important arguments to reduce further sealing and to protect urban green. However, the study responds to the increasing need to include the soil as an ecosystem service provider in further research as well as to detect complex connections between ecosystem service provision and land use change as part of urban land teleconnections. Nevertheless, this study showed that the basis for an ecological sustainable management of urban soil sealing steering is assured, especially by legal- and informal-planning strategies. However, since sealing is further increasing in Europe, it can be concluded that strategies lack efficient implementation. Therefore, further research should focus on assessing the steering potential of these responses (e.g., acceptance of responses, control of steering success) by integrating actors of the meso- and microscale (as the main steering addressees) into the assessment process.
